# Simultaneous Cooperation and Competition in the Evolution of Musical Behavior: Sex-Related Modulations of the Singer's Formant in Human Chorusing

**DOI:** 10.3389/fpsyg.2017.01559

**Published:** 2017-09-14

**Authors:** Peter E. Keller, Rasmus König, Giacomo Novembre

**Affiliations:** ^1^MARCS Institute for Brain, Behaviour and Development, Western Sydney University Sydney, NSW, Australia; ^2^Max Planck Institute for Human Cognitive and Brain Sciences Leipzig, Germany; ^3^Department of Neuroscience, Physiology and Pharmacology, University College London London, United Kingdom

**Keywords:** music, vocal expression, singer's formant, evolution, non-verbal communication

## Abstract

Human interaction through music is a vital part of social life across cultures. Influential accounts of the evolutionary origins of music favor cooperative functions related to social cohesion or competitive functions linked to sexual selection. However, work on non-human “chorusing” displays, as produced by congregations of male insects and frogs to attract female mates, suggests that cooperative and competitive functions may coexist. In such chorusing, rhythmic coordination between signalers, which maximizes the salience of the collective broadcast, can arise through competitive mechanisms by which individual males jam rival signals. Here, we show that mixtures of cooperative and competitive behavior also occur in human music. Acoustic analyses of the renowned St. Thomas Choir revealed that, in the presence of female listeners, boys with the deepest voices enhance vocal brilliance and carrying power by boosting high spectral energy. This vocal enhancement may reflect sexually mature males competing for female attention in a covert manner that does not undermine collaborative musical goals. The evolutionary benefits of music may thus lie in its aptness as a medium for balancing sexually motivated behavior and group cohesion.

## Introduction

Music is a social communicative art form whose pervasiveness across human cultures suggests convergent evolutionary origins (McDermott and Hauser, 2005; Fitch, 2006; Merker et al., 2015). Although, these origins remain as mysterious today as they were in antiquity (McDermott, 2008; Morley, 2013), the comparative study of communicative signaling in human and non-human species has identified two broad classes of factors—competitive and cooperative—that could have driven the evolution of musical behavior (Brown, 2000; Mithen, 2005; Cross and Morley, 2009; Hagen and Hammerstein, 2009; Merker et al., 2015).

Competition for mates and territory in a variety of non-human animal species (e.g., songbirds and humpback whales) has selected for music-like signaling behavior that increases an individual's prospects for reproduction and survival by attracting sexual partners and repelling rivals (Catchpole and Slater, 1995; Marler, 2000; Fitch, 2006; Smith et al., 2008). In the case of birdsong, playback experiments with European starlings, wrens, and flycatchers have shown that females are attracted to audio recordings of conspecific male vocalizations (Eriksson and Wallin, 1986; Mountjoy and Lemon, 1991; Johnson and Searcy, 1996). Related work with dunnocks has found that female responsiveness is greatest when the recording is of an alpha male, the song is complex, and the female is in her fertile period (Wiley et al., 1991). Furthermore, studies of song production in great reed warblers have demonstrated that males sing longer, more complicated songs when advertising for females than when already paired (Catchpole, 1983). Playback of such complex male songs elicits increased sexual display behavior in females (Catchpole et al., 1986), and males who sing more complex songs attract more females and produce more offspring (Catchpole, 1986). Song-like signaling may also play a role in attracting potential mates in some marine mammals. For example, the incidence of singing in male humpback whales is greater in the presence of unescorted mother-calf pairs (in which the adult female provides a mating opportunity) than for mother-calf pairs who are already escorted by another male (Smith et al., 2008). By analogy, it has been proposed—originally by Darwin (1871)—that the genesis of human music lies in courtship displays that evolved through sexual selection fuelled by competition between individuals (Miller, 2000).

Cooperative accounts provide an alternative by focusing on benefits to the individual gained through group membership. Group music making is a vital part of social life across the world's cultures, with individuals frequently coming together to communicate emotions and expressive intentions through rhythmically coordinated body movements and sounds (Merriam, 1964; Blacking, 1973; Nettl, 1983). In other social animals, the demands of cooperating with conspecifics has selected for collective displays where the inter-individual coordination of signals in space and time facilitates pair bonding [e.g., gibbon “duets” (Geissmann, 2000) and whistle matching in dolphins (Janik, 2000)] and group cohesion [e.g., call-and-response acoustic signaling in bats (Chaverri et al., 2012)]. Social accounts of the origins of human music hold that rhythmic coordination between individuals engaged in collaborative musical activities similarly facilitates cooperative behavior by promoting interpersonal bonding and feelings of mutual affiliation, trust, and commitment (Brown, 2000; Huron, 2001; Kirschner and Tomasello, 2010; Tarr et al., 2014; Weinstein et al., 2016). Practices involving interacting with others through music thus buttress human society and culture by exploiting the basic capacity for rhythmic interpersonal coordination to foster pro-social behavior (Lomax and Berkowitz, 1972; Mithen, 2005; Cross, 2012; Morley, 2013; Launay et al., 2016).

Accounts based on competition or cooperation alone, however, are not sufficient for dealing with the extensive palette of musical behaviors in the human repertoire, ranging from soothing lullabies, through festive folksongs and magnificent ceremonial and “art” music, to aggressive war dances and rap battles (Merriam, 1964; Nettl, 1983; Clayton, 2009; Clayton et al., 2012). An alternative is that human music serves both cooperative and competitive functions (Brown, 2000). On this view, music does not function solely in sexual or natural selection at the level of the individual or as a vehicle for group selection, but may rather play roles at both the level of the individual and the group.

The motivation for this unifying view comes from multiple angles. To begin with, it has been argued that sexual selection alone is not viable because music is rarely used as an obvious courtship display and is not a sexually dimorphic trait in humans (Brown, 2000). Natural selection is more generally problematic because the precise sense in which music serves to adapt individuals to their environment, and thereby enhance one's chance of survival, is obscure (Fitch, 2006). It has, however, been countered that pure cooperative accounts are questionable because they rely on group selection, which some evolutionary theorists consider to be a weaker explanatory principle than individual selection (Miller, 2000).

To circumvent these drawbacks, unifying accounts of human musical behavior postulating multilevel selection of both cooperative and cooperative functions have been advanced. One such account holds that coordinated displays in music and dance are beneficial in communicating information about coalition quality within a group to potential allies and competitors outside the group (Hagen and Bryant, 2003). Another unified account posits that music can function as a reward system that reinforces vital ritualistic group behaviors under some circumstances and as courtship or fitness displays in others (Brown, 2000). A strong version of the hypothesis that music functions both cooperatively and competitively, which we advance here, is that music is capable of doing so simultaneously, thus supporting different forms of communication in parallel at the level of the group and the individual. This dual-function hypothesis is motivated by work on rhythmically coordinated communal displays in non-human species (Merker, 2000).

Although, group music making can be viewed as an exalted human activity, it is only one amongst several stunning examples of rhythmic communal signaling in nature (Bailey, 1991; Merker, 2000; Ravignani et al., 2014; Merker et al., 2015). Bioluminescent fireflies flash together (Buck, 1988; Branham and Greenfield, 1996), fiddler crabs collectively wave their oversized claws (Murai and Backwell, 2006), fish drum their swim bladders in concert (Locascio and Mann, 2011), and cicadas (Hoppensteadt and Keller, 1976), crickets (Walker, 1969; Snedden and Greenfield, 1998), and frogs (Whitney and Krebs, 1975; Partridge and Krebs, 1978) produce choruses of sounds wherein coordination ranges from strict synchrony to relatively flexible alternation (Greenfield, 2005; Ravignani et al., 2014).

These non-human displays, which are typically produced by congregations of males, fulfill functions related to courtship and conflict avoidance (Greenfield, 1994). By coordinating their signals, group members maximize the amplitude of their collective broadcast and thereby increase its salience and geographic reach, resulting in a “beacon” effect (Buck and Buck, 1966) that enhances the ability to attract distant females, repel territorial adversaries, and confuse predators (Greenfield, 2005). While such coordinated behavior appears cooperative, research on communal sexual displays suggests that, in a number of species, it arises from the operation of competitive mechanisms by which individual males jam—i.e., block or interfere with—rival signals (Whitney and Krebs, 1975; Greenfield and Roizen, 1993; Greenfield, 1994).

On this account, males who signal faster than their neighbors when competing for female attention gain an advantage by producing relatively early signals that effectively mask following signals (Otte and Loftus-Hills, 1979; Greenfield, 1994). Precise temporal coordination between males nevertheless ensues when leading calls trigger a call in neighbor after a fixed phase delay or a rebound interval associated with inhibitory resetting of a neural pacemaker (Buck, 1988; Greenfield and Roizen, 1993), with globally stable patterns of interaction occurring when variations in individual signaling rates cause switches in momentary leadership (Greenfield et al., 1997). Group coordination under such circumstances is thus an epiphenomenon resulting from competitive mechanisms selected by general female preferences for male signals that contain greater energy due to delivery at a fast rate or high intensity (Greenfield, 2005).

The current case study tested the hypothesis that human music is likewise capable of simultaneously serving cooperative and competitive functions. To this end, we investigated the effect of introducing females into an otherwise male audience during repeated performances of a short concert performed by an internationally acclaimed male choir, the St. Thomas Choir of Leipzig, famed for its 800-year tradition of excellence under directors including J. S. Bach. Consistent with the dual-function hypothesis, it was expected that the presence of females might elicit vocal embellishments that allow individual males to compete for female attention in a manner that does not undermine ensemble cohesion. Moreover, this effect should be most prevalent in sexually mature males who have already undergone puberty-related maturational changes that deepen the voice, that is, in members of the tenor and bass sections of the choir.

To detect the anticipated vocal embellishments, we recorded individual singers from the choir and then conducted acoustic analyses of spectral energy, sound intensity, performance tempo, and tone onset timing. It was expected that spectral energy (which affects vocal tone quality) would be a likely locus of effects of female presence, as vocal spectrum can be varied without influencing performance parameters such as tuning and timing that could compromise ensemble balance and coordination. Furthermore, aspects of performance that affect ensemble cohesion in terms of timing and balance may vary across repeat performances due to practice effects. To take these effects into account, we measured the degree of tempo matching across parts, interpersonal asynchrony, and balance in terms of relative loudness. Modulations of these indices of ensemble cohesion were not expected to occur due to the extensive experience of the St. Thomas Choir with group performance in general and also with the specific pieces that were sung.

Instead, we were particularly interested in a high frequency band of voice's spectrum, known as the “singer's formant” (~2,500–3,500 Hz). Increased energy in the singer's formant adds carrying power and brilliance to the voice by emphasizing a frequency region to which human hearing is maximally sensitive (Sundberg, 1974, 1999). In accompanied solo singing, a prominent singer's formant assists the voice to stand out from background instrumental sounds, thus allowing opera singers to project their voices above loud orchestras, which contain relatively little energy in this frequency region (Sundberg, 1987). The use of the singer's formant in choral singing is a matter of debate. Although, enhancement of the singer's formant is not typically advocated in choral singing (where the focus is on the blending of voices; Ternström, 1991, 2003), it can nevertheless be observed in choral performance under some circumstances (especially when the singers are also experienced soloists; Rossing et al., 1986).

## Materials and methods

### Participants: the St. Thomas Choir

Sixteen members of the St. Thomas Choir of Leipzig, a boys' choir in Germany, participated in the study (*N* = 16): Four sopranos, four altos, four tenors, and four basses. Sopranos were aged 12 (*n* = 2) and 13 years (*n* = 2); altos were aged 12 (*n* = 2), 13 (*n* = 1), and 16 years (*n* = 1, singing falsetto); tenors were aged 16 (*n* = 2) and 18 years (*n* = 2); basses were aged 16 (*n* = 1), 17 (*n* = 2), and 19 years (*n* = 1). All singers were naïve to the purpose of the study. A senior member of the choir (a tenor, aged 18 years) who held the position of prefect and was also naïve to the purpose of the study served as the conductor during the recording session.

All participants had thorough musical training, high levels of vocal skill, and extensive experience singing together. The St. Thomas Choir, which is an acclaimed ensemble that rehearses daily, tours globally, produces commercial recordings, and maintains a schedule of three performances per week that attract crowds of tourists to the St. Thomas Church in Leipzig: http://www.thomanerchor.de. The choir was established in 1212 and has run continuously since then under the leadership of esteemed cantors (musical directors), including Johann Sebastian Bach. Members of the choir all attend the St. Thomas School, a public boarding school, from the fourth to twelfth class. The music training that choir members receive during this period includes weekly singing and instrumental lessons, music theory classes, instruction in phonation, and daily individual practice sessions and rehearsal of the choir in full and in sections.

At the time of data collection for the current study, the St. Thomas Choir included 103 members in total. The 16 students who participated in our study were recruited following earlier consultation with the cantor's assistant, who was asked to recommend the best available choristers. Thus, although the participant sample is small, it comprises elite individuals with highly honed vocal and ensemble skills. Participants' parents provided written informed consent for their sons to take part in the study, which was run in accordance with the declaration of Helsinki and protocol approved by the ethics committee of the University of Leipzig.

### Materials

The materials consisted of two distinctive pieces of choral music composed by Johann Sebastian Bach. One (a chorale) requires strict synchrony between soprano, alto, tenor, and bass sections of the choir and the other (a fugue) is characterized by greater rhythmic independence between voices. Both pieces are in the standard repertoire of the St. Thomas Choir, and all participants were familiar with the pieces and had sung them on numerous previous occasions.

The chorale “Du heilige Brunst, süßer Trost [You holy zeal, sweet consolation]” is from the motet “Der Geist hilft unser Schwachheit auf [The Spirit gives aid to our weakness]” (BWV 226) composed by Bach in Leipzig in 1729. The chorale setting is 24 bars long, in quadruple meter (with 4 quarter-note beats per bar), homophonic in texture (although parts contain passing notes), and, with its stately rhythm and holy text (from a Pentecostal hymn written by Martin Luther), may be described as “reverent” in character. The fugue comes from the motet “Singet dem Herrn ein neues Lied [Sing unto the Lord a new song]” (BWV 225), first performed in Leipzig around 1727. The fugue—a setting of biblical text “Alles, was Odem hat, lobe den Herrn [All that have voice, praise the Lord]” from Psalm 150:6—spans 112 bars (from bar 256 to the end of the work), is in triple meter (with 3 eighth-note beats per bar), polyphonic in texture, and has a “lively” character featuring rapid runs of sixteenth notes cascading through the choral parts.

Both pieces are written in the key of B-flat major with four vocal parts: soprano, alto, tenor, and bass. For the chorale, the soprano part contains 95 notes ranging in pitch from F4 to G5 (349–784 Hz), the alto part has 104 notes ranging from C4 to D5 (262–587 Hz), the tenor part has 110 notes ranging from E-flat3 to F4 (156–349 Hz), and the bass part has 120 notes ranging from B-flat2 to D4 (117–294 Hz). For the fugue, the soprano part has 320 notes (F4 to B-flat5; 349–932 Hz), the alto part has 334 notes (B-flat3 to D5; 233–587 Hz), the tenor part has 369 notes (F3 to A5; 175–440 Hz), and the bass part has 419 notes (G2 to E-flat4; 98–311 Hz).

### Procedure

The study took place in the chamber music auditorium at Castle Colditz in Germany, where the St. Thomas Choir was interned at their annual summer camp. The auditorium contained a stage area, which was large enough to accommodate the choir comfortably, and six rows of tiered seating. The procedure was completed in a single 1-h session. Prior to commencing the session, the 16 members of the choir and the conductor were assembled and introduced to the researchers. They were told that the researchers were studying choral singing and were specifically interested in the level of balance, timing precision, and intonation achieved in a top choir. Members of the choir and the conductor were given the musical scores for the two pieces and were told that the upcoming task involved singing three repeat performances of the pieces.

Choir members were then fitted with individual head-worn wireless microphones (Sennheiser ME 3). Signals picked up by the microphones were sent via radio transmitters (Sennheiser SK 500) to a receiver (Sennheiser EW 500) connected to a stage unit (Roland S-1608) with built-in analog-to-digital converter and microphone preamplifiers. The stage unit routed the audio to a digital mixer (Roland M-300), which was used to record each singer's voice onto a separate channel with digital audio software (Cakewalk Sonar X1 Producer) running on a laptop computer (Dell Inspiron with REAC driver). The equipment was configured to record audio in each channel in mono at a sampling rate of 48 kHz in .WAV format. In addition to this multi-track audio recording, two digital video cameras (Sony HDR-HC7) were used to record the performances. One camera was positioned at the rear of the auditorium and was oriented in such a way that it captured a frontal view of the choir. The second camera, which was placed to the side of the choir, recorded a frontal view of the audience.

Once the audio set up was complete, choir members were asked to stand in two rows, as per their conventional formation, with the front row consisting of the four sopranos on the left and the four altos on the right, and the back row consisting of the four tenors on the left (behind the sopranos) and the four basses on the right (behind the altos). The conductor was positioned in front of the choir, facing the singers (and could therefore not see the audience while conducting). When in position, a sound check was performed to verify that each channel was receiving a clear signal and to optimize recording levels. The conductor was then asked to run through each piece once with the choir in preparation for the recording session.

The choir was informed that there would be researchers around throughout the recording session, as well as a small audience to create a natural performance situation. The choir was also told that a “school group” was due to arrive for a castle tour and would join the audience for a performance and would be taking notes for a school magazine article. This school group consisted of four females who constituted our manipulation (described below). The choir was instructed to sing as they normally would in a concert performance.

In the recording session, the choir performed the two pieces, one after the other, three times each in a separate “concert.” Recordings of the performances are provided as Supplementary Audio. The chorale was performed before the fugue in each concert. Each concert was separated by an intermission lasting 1–3 min, during which the composition of the audience was changed. The first of the three concerts was sung to a small audience comprised of adult male listeners. Four adolescent females were added to the audience for the second concert (which also included the males). The females then departed before the third concert, which was sung to the original male audience. (While we would have liked to vary the type of additional audience members systematically in a larger study design, including younger and older females and males of corresponding ages, such extra manipulations were precluded by the limited availability of the St. Thomas Choir).

The male audience members were four staff members of the St. Thomas School (aged between 35 and 55 years). Also present in the auditorium were two adult male researchers (aged 28 and 42 years), two adult female research assistants (aged 26 and 24 years), and an adult male recording engineer (aged 27 years) and photographer (aged 28 years). The adolescent female audience members were aged 15 (*n* = 2) and 16 years (*n* = 2). These females were relatives of one of the female research assistants, who invited them to attend the recording session. The females were naïve with regard to the goals of the study. Prior to entering the auditorium, each female was given a notepad and pen and instructed to make a written record of their impressions of the choir's performance during the concert. This task was intended to occupy the females and decrease the likelihood that they would engage in behavior that could possibly distract the choir. Examining the video recordings revealed no such behavior during the performances.

At the conclusion of the recording session, each member of the choir completed a questionnaire to evaluate his subjective impressions of the performances and views on choral singing. Questionnaire items addressed (1) the level of concentration achieved while singing, (2) the focus of attention (on self, neighboring singers, whole choir, conductor, and audience), (3) the degree to which the audience is focused upon generally in concerts, (4) the degree to which the audience proved distracting during the recording session, (5) whether the presence of girls in the audience led to changes in the individual's own performance, and (6) which of the three concerts went the best (see **Appendix**). Item 5, on effects of the girls, included several options that could be selected, including one that directly probed whether the respondent attempted to attract the girls' attention. The conductor was asked which performance he thought went the best.

### Data analysis

Prior to analysis, the audio recordings of individual voices for each performance of each piece were extracted from the continuous recordings of the full session. The extraction process involved opening the recordings of individual voices simultaneously in separate channels in Cubase LE 6 digital audio software, and then cutting the 16 tracks in such a way that they all started 1 s prior to the onset of the first vocal sound and ended 1 s after the offset of the final vocal sound in each performance for each piece. These individual voice recordings, thus synchronized across choir members, were then subject to acoustic analysis using MIRtoolbox 1.6.1 (Lartillot et al., 2008), a Matlab toolbox for the extraction of musical features from audio files. The following features were analyzed: energy in the singer's formant spectral region (2,500–3,500 Hz), sound intensity (to assess ensemble balance), global performance tempo, variability in local tempo, and relative note-onset timing (to assess the accuracy and precision of ensemble coordination). This latter analysis also made use of a full choir version for each performance created by mixing the 16 individual voice channels into a single mono file in .WAV format. The feature extraction process is described below.

#### Spectral energy

For data visualization, the spectral envelope was extracted from each individual voice file using the “mirspectrum” function of MIRtoolbox to decompose the signal into its frequency components via Fast Fourier Transform, and then using the “mirenvelope” function to fit a curve tracing the amplitude of each frequency component of this spectrum. The “Terhardt” option was used with “mirspectrum” to modulate the energy following the Terhardt (1979) outer ear model, which emphasizes frequencies around 2,000–5,000 Hz, at which human hearing is particularly sensitive.

For statistical analysis, the proportion of total energy in the 2,500–3,500 Hz spectral region of each individual voice file was computed using the “mirbrightness” function of MIRtoolbox. This function calculates the proportion of energy (0–1) above a user-specified cut-off frequency. We applied the “mirbrightness” function twice to each file, once with a cut-off frequency of 2,500 Hz and then with a cut-off frequency of 3,500 Hz, and subsequently computed the difference to yield the proportion of total energy between 2,500 and 3,500 Hz. A proportional measure was chosen because the relative amount of energy in high frequency regions is a reliable correlate of perceived timbre in vocal expression (von Bismarck, 1974; Laukka et al., 2005).

#### Sound intensity

Global sound intensity was measured by calculating the root mean square of the amplitude profile of each individual voice file using the “mirrms” function from MIRtoolbox. This analysis takes into account energy at all frequencies of the sound spectra, and therefore allows overall ensemble balance in terms of relative intensity of different voice parts to be assessed. The ANOVAs on these data allowed us to test whether female presence affected ensemble balance by changing intensity in some voices relative to others. This could occur if, for example, distraction caused by female presence disturbed the so-called “self-to-other ratio,” which reflects the degree to which an individual can hear their own sounds amongst co-performers' sounds (Ternström, 2003).

#### Global and local tempo

Global tempo was estimated for each individual voice file using the “mirtempo” function in MIRtoolbox. This function detects note onsets based on the amplitude envelope of the audio signal, then searches for periodicities in the note-onset time series for a range of tempi (40–200 beats-per-minute [bpm]), and finally selects the tempo yielding the maximum periodicity score. Variability in local tempo was assessed by calculating the standard deviation of time points in the tempo map for each file. The “Metre” option of “mirtempo” was employed in this analysis to take into account variability at metrical levels other than the beat period (Lartillot et al., 2013).

#### Relative note-onset timing

Note-onset times were estimated for each individual voice file, as well as for the mixed full choir file for each performance, using the “mironsets” function in MIRtoolbox. This function applies a peak-picking algorithm to an onset detection curve computed based on the audio signal's amplitude envelope. The “SpectroFrame” option was used in this analysis, with the frame length and hop factor both set to 150 ms to avoid the detection of spurious onsets. This value was chosen based on the assumption that, given the notated rhythms in the pieces and the tempi at which they were performed, successive note onsets would be separated by at least 150 ms.

To assess ensemble coordination, asynchronies between note onsets in each individual voice file and onsets in the mixed full choir file were computed for each performance. These asynchronies represent temporal deviations of estimated note onsets produced by each singer from estimated onsets in the choir's collectively produced sound, which served as a temporal frame of reference for the current analysis. Asynchrony series were generated with an algorithm that, for each note onset in a given individual voice file, searched for the nearest onset in the mixed full choir file and, if these two onsets were separated by <150 ms, recorded the asynchrony. Median asynchrony was taken as a measure of synchronization accuracy and the standard deviation of the asynchronies series was taken as a measure of synchronization precision for each asynchrony series.

#### Statistical tests

Data for spectral energy were entered into a (2 × 2) × 4 mixed Analysis of Variance (ANOVA) with female presence (present, absent) and piece (chorale, fugue) as within-participant factors, and voice type (bass, tenor, alto, soprano) as a between-participants factor. The criterion for statistical significance was set at α = 0.05 in all analyses reported here. Significant interaction effects were followed up with two-tailed paired-samples *t*-tests comparing spectral energy when females were present vs. absent for each voice type separately. Given the stylistic differences between the two pieces, affecting musical texture, dynamics, rhythm, and tempo, data for each of the remaining features were entered into a separate (2) × 4 ANOVA, with factors female presence (present vs. absent) and voice type (bass, tenor, alto, soprano), for each piece.

## Results and discussion

The results reported below address the effects of the presence of female audience members on individual voices recorded from the soprano, alto, tenor, and bass sections of the St. Thomas Choir during performances of two distinctive musical pieces. Results for spectral energy in individual boys' voices (focusing on 2,500–3,500 Hz “singer's formant” region) are presented first, followed by sound intensity (to assess ensemble balance), global performance tempo, variability in local tempo, and relative tone-onset timing (to assess the accuracy and precision of ensemble coordination).

### Spectral energy

**Figure 1:**
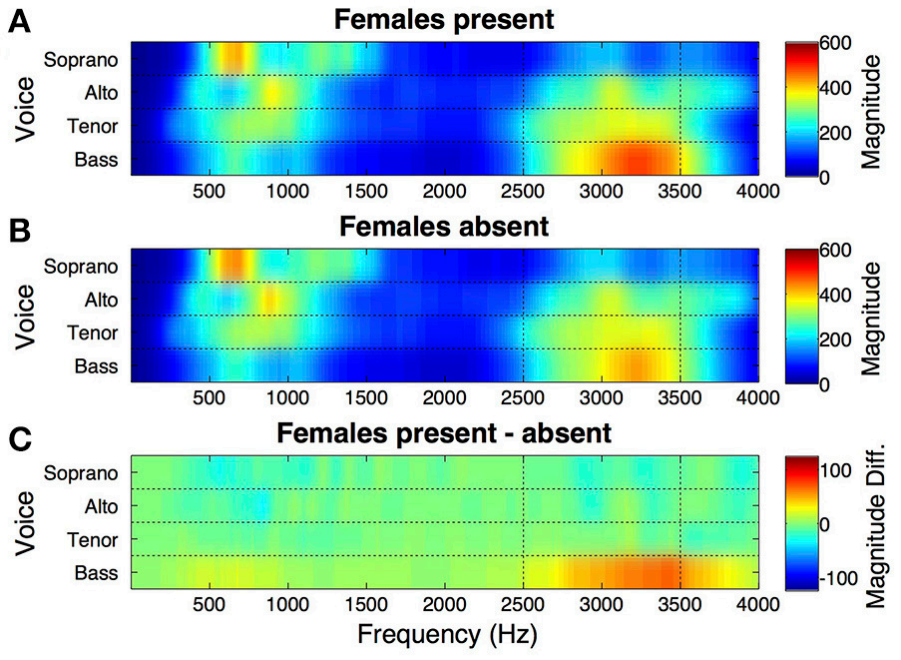
Effect of female presence on spectral energy in male voices singing in chorus. **(A)** Time-averaged spectra for choir voice sections singing with adolescent females present in the audience. Output of the Terhardt outer ear model (see Section Spectral Energy) is shown for audio signal inputs at frequencies up to 4,000 Hz in sopranos, altos, tenors, and basses. Audio was recorded with individual head-worn microphones as the choir performed two distinctive pieces. Mean spectral magnitude is shown for each voice section (4 singers per section), averaged across the two pieces. There is a relatively high peak in energy in the singer's formant region (2,500–3,500 Hz) for basses. **(B)** Average spectra for voices when singing the same pieces to a male audience with females absent. The energy peak in basses in the singer's formant region is weaker than when females were present. **(C)** The difference between spectral magnitudes when females were present vs. absent, highlighting the singer's formant increase in basses.

Spectral energy was computed for individual singers from audio signals for each of performance of each piece. Time-averaged mean spectral magnitude for sopranos, altos, tenors, and basses (4 singers per part), averaged across the two pieces (chorale and fugue), for performances with female audience members present vs. absent are presented in Figure 1. For display, the plot shows output of the Terhardt (1979) outer ear model for audio signal inputs at frequencies up to 4,000 Hz. Here it can be seen that there is a relatively high peak in energy in a high frequency band (~2,500–3,500 Hz, marked by vertical dotted lines) of the vocal spectrum, corresponding to the singer's formant, in the bass section.

**Figure 2:**
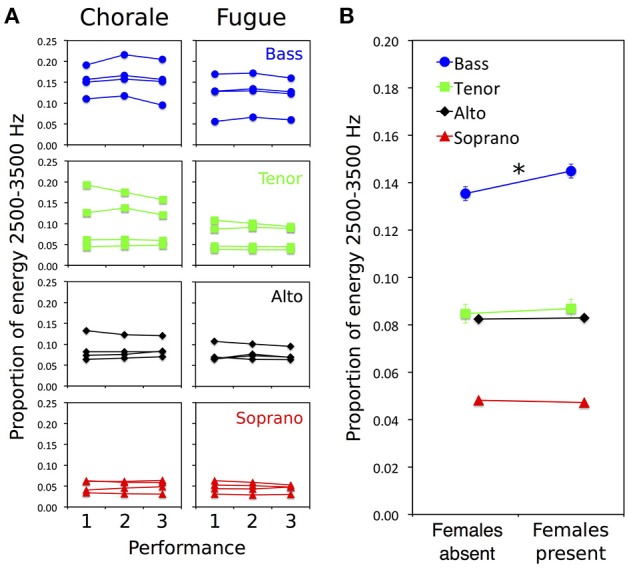
Effects of female presence on spectral energy (based on audio signals) in the voices of individual male singers for two distinctive pieces of choir music. **(A)** Proportion of energy in the singer's formant region for individual singers in each voice section for three performances of each piece, a chorale (left) requiring rhythmic synchrony between choir sections and a fugue (right) requiring rhythmic independence between sections. Performance 1 was sung to the male audience, performance 2 to the audience with additional females, and performance 3 to the male audience again. Separate plots are shown for basses (blue circles), tenors (green squares), altos (black diamonds), and sopranos (red triangles). For both pieces, there is an increase in energy from performance 1 to 2 (when females present), followed by a decrease in performance 3, for each of the four basses, but no such consistent effect in other voice sections. **(B)** Proportion of total energy in the singer's formant region for each voice part (averaged across individual singers and pieces) when females were present vs. absent. Proportion of energy is higher when females were present than absent only in basses (female presence × voice type interaction, *p* = 0.003; asterisk indicates *p* = 0.016 for simple effect of female presence for basses only). Error bars are 95% confidence intervals based on within-participants s.e.m. (bars occluded by data marker symbols for sopranos and altos).

For statistical analysis, the proportion of total energy in the 2,500–3,500 Hz singer's formant region of each individual voice file was calculated (on the raw audio signal, without applying the Terhardt model). Figure 2A shows the proportion of energy in this region for individual singers in each voice section for the three performances of the two pieces. Performance 1 was sung to the male audience, performance 2 to the audience with additional females, and performance 3 to the male audience again. For both pieces, there is an increase in energy from performance 1 to 2 (when females were present), followed by a decrease in performance 3, for each of the four basses, but no such consistent pattern in other voice sections. Figure 2B highlights the main result by displaying the proportion of total energy in the singer's formant region for each voice part (averaged across individual singers and pieces) when females were present vs. absent.

An ANOVA testing for effects of female presence (present, absent), piece (chorale, fugue), and voice type (bass, tenor, alto, soprano) on proportion of energy in the singer's formant range revealed statistically significant main effects of female presence [*F*_(1, 12)_ = 15.191, *p* = 0.002], piece [*F*_(1, 12)_ = 25.180, *p* < 0.001], and voice type [*F*_(3, 12)_ = 5.634, *p* = 0.012], as well as a significant interaction between female presence and voice type [*F*_(3, 12)_ = 8.440, *p* = 0.003]. The remaining interaction effects were not statistically significant (*p*s > 0.05).

The main effect of female presence indicates that energy in the 2,500–3,500 Hz singer's formant region was generally higher when females were present than absent. This effect was, however, qualified by the interaction between female presence and voice type, suggesting that—as can be seen in Figure 2B—the bass section was primarily responsible the increase in energy observed when females were present. Paired-samples *t*-tests confirmed that the effect of female presence was statistically significant for the basses [*t*_(3)_ = 4.917, *p* = 0.016] while reliable effects were not observed for tenors (*p* = 0.462), altos (*p* = 0.487), or sopranos (*p* = 0.554).

The specificity of the effect to members of the bass section, that is, to singers with the deepest voices, is noteworthy. A prominent singer's formant is conventionally considered to be more desirable in solo than choral singing (Ternström, 2003). However, because enhancing the singer's formant does not affect other performance parameters such as tuning and timing, the observed effect may reflect sexually mature males competing for female attention in a manner that does not threaten group cohesion.

The main effect of piece indicates that proportion of energy in the 2,500–3,500 Hz range was generally greater for the chorale than the fugue, which may be due to differences in compositional texture and rhythmic structure between the pieces. Specifically, enhancement of the singer's formant might have been elicited by the homogenous texture of the chorale, where separate voice sections sing predominantly in rhythmic unison, more so than for the polyphonic fugue, where the vocal lines sung by separate voice sections are differentiated in terms of rhythm. Furthermore, longer note durations in the chorale (mainly quarter and eighth notes at a steady tempo) than the fugue (eighth and sixteenth notes at a rapid tempo) may generally allow more scope for modulating spectral properties of vocal sound.

The main effect of voice type indicates that singer's formant energy increased across choir sections from sopranos through altos and tenors to basses. This increase may be due in part to differences in the perceptual salience of separate voice parts, as well as to changes in vocal tract size and shape associated with maturational development. With regard to perceptual salience, the general tendency for superior processing of high voices in multi-part musical textures (Palmer and Holleran, 1994; Crawley et al., 2002; Trainor et al., 2014) implies little need to employ additional techniques to enhance the soprano part. With regard to vocal tract configuration, the lowering of the larynx and widening of the pharynx during adolescent development (Fitch and Giedd, 1999) increases the size of a resonance cavity that causes clustering of high formants in the voice spectrum (Sundberg, 1987). Boys who have undergone growth associated with puberty are therefore more likely to display a prominent singer's formant and, in adults, enhancement of the signer's formant is more commonly observed in male tenors and basses than in female sopranos and altos (Sundberg, 1999). Given that participant age was related to voice type (though note that tenors and basses were similar in age), it is not surprising that energy in the 2,500–3,500 Hz region increased across voice types.

It can also be noted that age differences were not responsible for the presence of the effect in basses and absence of the effect in tenors. Although, two tenors exhibited an enhanced singer's formant during the second performance (see Figure 2A), these individuals were in fact younger (aged 16 years) than the other two tenors who did not show an effect (aged 18 years).

### Sound intensity

Global sound intensity was measured by calculating the root mean square of the amplitude profile of each individual voice file. Average data for each voice section for each performance of each piece are shown in Table 1. Separate ANOVAs, testing for effects of female presence (present vs. absent) and voice type (bass, tenor, alto, soprano) on sound intensity, for each piece yielded no statistically significant effects (*p*s > 0.05). The absence of significant interactions involving voice type suggests that ensemble balance, in terms of relative intensity of different voice parts, was consistent across performances.

**Table 1:** Measures of sound intensity (root mean square of amplitude profile), global tempo (bpm), local tempo variability (SD of onsets in tempo map), median asynchrony (in ms, relative to note onsets in mixed full choir file), and SD of asynchronies (ms) computed from individual audio files for three performances (females present in audience for performance 2) of two pieces (chorale and fugue).

**Measure**	**Voice**	**Chorale**						**Fugue**					
		**Performance**						**Performance**					
		**1**		**2**		**3**		**1**		**2**		**3**	
Sound intensity	Soprano	0.249	(0.056)	0.231	(0.045)	0.246	(0.057)	0.242	(0.051)	0.242	(0.047)	0.258	(0.056)
	Alto	0.207	(0.024)	0.171	(0.017)	0.171	(0.018)	0.255	(0.017)	0.237	(0.009)	0.226	(0.009)
	Tenor	0.184	(0.019)	0.180	(0.019)	0.169	(0.015)	0.254	(0.019)	0.256	(0.018)	0.248	(0.017)
	Bass	0.106	(0.009)	0.117	(0.015)	0.106	(0.010)	0.152	(0.014)	0.179	(0.025)	0.163	(0.018)
Global tempo	Soprano	83.84	(1.70)	80.76	(1.52)	84.98	(1.23)	161.40	(1.86)	162.51	(0.51)	161.98	(0.48)
	Alto	77.66	(0.85)	81.21	(1.29)	81.00	(2.68)	158.91	(0.98)	160.85	(0.92)	160.05	(1.20)
	Tenor	75.74	(2.47)	77.98	(0.81)	81.59	(1.31)	158.75	(1.62)	160.74	(0.72)	158.00	(1.80)
	Bass	81.97	(2.24)	78.31	(1.43)	82.22	(2.90)	160.32	(0.58)	163.63	(0.88)	162.23	(1.06)
Local tempo	Soprano	6.46	(0.94)	7.78	(1.87)	4.90	(1.13)	10.33	(3.14)	6.17	(1.55)	9.23	(2.24)
	Alto	20.20	(6.89)	10.14	(2.70)	10.58	(4.20)	24.27	(10.10)	19.16	(6.99)	13.00	(1.70)
	Tenor	6.23	(2.04)	10.34	(1.97)	10.89	(2.25)	7.61	(2.20)	9.14	(3.61)	9.55	(1.96)
	Bass	4.02	(0.16)	14.61	(11.19)	9.29	(2.33)	13.91	(5.43)	8.98	(2.59)	10.33	(5.30)
Median asynchrony	Soprano	−5.02	(4.48)	−0.70	(4.69)	−7.15	(6.79)	−3.26	(9.48)	−2.78	(8.81)	−7.07	(8.45)
	Alto	−4.75	(3.96)	−3.21	(4.60)	2.30	(2.43)	−5.84	(3.11)	−3.94	(2.52)	−1.88	(3.08)
	Tenor	7.96	(4.12)	−0.11	(2.72)	0.80	(3.00)	0.79	(5.75)	−0.77	(4.78)	−1.64	(5.06)
	Bass	4.15	(5.58)	8.19	(1.83)	1.44	(2.59)	−0.96	(4.34)	4.01	(4.31)	−0.89	(4.28)
SD of asynchronies	Soprano	61.23	(2.55)	59.01	(3.05)	57.03	(2.42)	45.81	(0.67)	46.75	(1.69)	45.11	(1.14)
	Alto	59.14	(1.46)	57.24	(1.45)	56.49	(0.69)	47.70	(1.66)	46.09	(1.36)	48.23	(1.69)
	Tenor	59.48	(1.82)	58.58	(0.95)	61.42	(1.96)	48.89	(1.31)	49.75	(1.61)	48.45	(1.84)
	Bass	59.15	(2.79)	60.60	(1.30)	58.19	(2.10)	53.15	(0.89)	51.13	(1.40)	49.97	(1.31)

*See Data Analysis for details. Values averaged across the four singers in each voice section (soprano, alto, tenor, and bass; s.e.m. in parentheses)*.

### Global and local tempo

Global tempo was estimated for each individual voice file and data for this measure are displayed in Table 1. The average estimated global tempo across voices was 80.34 bpm for the chorale and 161.07 bpm for the fugue. An ANOVA on global tempo data for the chorale revealed a statistically significant main effect of female presence [*F*_(1, 12)_ = 5.357, *p* = 0.039] and a significant interaction of female presence and voice type [*F*_(3, 12)_ = 4.009, *p* = 0.034]. The main effect of voice type was not significant. These results reflect an overall slightly *slower* performance tempo when females were present (81.12 bpm) than absent (79.56 bpm), with this effect being more pronounced in sopranos and basses than in altos and tenors (although *t*-tests for each voice type failed to reveal any significant effects). In contrast, an ANOVA on tempo data for the fugue only yielded a significant main effect of female presence [*F*_(1, 12)_ = 13.453, *p* = 0.003], reflecting overall slightly *faster* performance tempo when females were present (161.93 bpm) than absent (160.20 bpm). The effects of female presence on global tempo were therefore weak and inconsistent across the two pieces. Variability in local tempo was assessed by calculating the standard deviation of time points in a tempo map for each file. The ANOVAs on local tempo variability data (Table 1) yielded no statistically significant effects for either piece (*p*s > 0.05).

### Ensemble coordination

To assess ensemble coordination, asynchronies between note onsets in each individual voice file and onsets in the mixed full choir file were computed for each performance. These asynchronies represent temporal deviations of estimated note onsets produced by each singer from estimated onsets in the choir's collectively produced sound. The median of each individual singer's asynchrony series was calculated as a measure of his synchronization accuracy and the standard deviation of the asynchrony series was taken as a measure of synchronization precision (Table 1; Rasch, 1988).

The sign of the median asynchrony (negative or positive) is informative about whether note onsets in a particular voice part occur relatively early or late. In the current performances, the sopranos showed a numerical tendency to lead (median asynchrony = −4.33 ms, averaged across the four singers), followed by altos (−2.89 ms), tenors (1.17 ms), and basses (2.66 ms). These differences were, however, not statistically significant. ANOVAs on synchronization accuracy did not reveal significant effects for either piece (*p*s > 0.05). With regard to synchronization precision, the mean standard deviation of asynchronies, averaged across all singers, was 59 ms for the chorale and 48 ms for the fugue, which is commensurate with levels typically observed in expert ensembles (Keller, 2014). ANOVAs on synchronization precision did not yield statistically significant effects (*p*s > 0.05), apart from a main effect of voice type in the fugue [*F*_(3, 12)_ = 3.961, *p* = 0.036], reflecting a general decrease in precision from sopranos to basses (which may be a consequence of the increase in number of notes across these parts; see Section Materials).

### Subjective evaluations

After the recording session, each member of the choir completed a questionnaire addressing his evaluation of the performances and views on choral singing in general. Analysis of the questionnaire items revealed no reliable differences between voice types in terms of (1) self-reported level of concentration, (2) focus of attention on self, neighboring singers, whole choir, conductor, or audience, (3) general tendency to focus on the audience during concerts, (4) the degree to which the audience proved distracting during the recording, (5) whether the presence of girls led to changes in the individual's own performance. For this last item, none of the boys selected the response option indicating that they attempted to attract the girls' attention. In response to a final item probing views on which of the three concerts went the best, all tenors and basses thought that the one sung in the presence of females was best, while only one out of the four sopranos and no altos thought that this was the case. The conductor (who could not see the audience while conducting) indicated that the performance with females present went the best.

## General discussion

The present case study tested the hypothesis—motivated by research on non-human “chorusing” displays (such as those produced by congregations of male insects and frogs to attract females)—that group music making serves cooperative and competitive functions simultaneously. In support of this dual-function hypothesis, acoustic analyses of individual voices from the St. Thomas Choir of Leipzig revealed increased energy in the singer's formant spectral region (2,500–3,500 Hz) in members of the bass section, that is, boys with the deepest voices, when females were included in an otherwise male audience during repeated performances of a concert. This selective effect of female presence generalized across different musical pieces—a chorale requiring synchrony between sections of the choir and a fugue characterized by greater rhythmic independence between voices—and did not disrupt ensemble cohesion in terms of balance or temporal coordination. Enhancing the singer's formant generally modifies vocal tone quality by imbuing the voice with what Helmholtz (1875, p. 116) described as “a clear tinkling of little bells”. Observing such subtle embellishment in the context of choral singing suggests that, under conditions where group cohesion is strongly favored, sexually mature males have at their disposal a covert means to compete for female attention.

Evidence that the enhanced singer's formant was motivated by sexual competition lies in the finding that the effect of female presence was only reliably observed in members of the bass section of the choir. The presence of adolescent females (aged 15–16 years) may have elicited competitive behavior exclusively in bass singers (aged 16–19 years) due to relatively high levels of the sex hormone testosterone in males with the deepest voices (Zitzmann and Nieschlag, 2001). Testosterone lengthens the vocal tract by stimulating a secondary descent of the larynx in males during puberty (Fant, 1975). This lengthening lowers the fundamental frequency of the voice and reduces dispersion in formant frequencies (Bruckert et al., 2006), which increases perceived attractiveness and dominance (Evans et al., 2008). The boost in the singer's formant that we observed may constitute an attempt by the basses to establish a privileged social communication channel with female listeners by drawing attention to these appealing vocal qualities.

The fact that female presence did not lead to a reliable enhancement of the singer's formant in tenors (aged 16–18 years) suggests that the effect was not related to a general desire to lift performance standard in the presence of female peers. An increase in the desire to perform well could also be expected to affect other performance parameters, even in sopranos (12–16 years) and altos (12–16 years), but no such additional effects were observed. Furthermore, as revealed by post-concert questionnaire responses, members of the tenor and bass section were unanimous in thinking that the performance sung in the presence of females was the best, but none admitted to attempting to attract the girls' attention. Therefore, while tenors and basses converged in terms of their subjective evaluations of the performances, their objectively measured behavior diverged.

Our results point to an analogy between human choral singing and chorusing displays in non-human species (crickets, cicadas, and frogs) where congregations of males produce rhythmically coordinated signals that collectively serve as a beacon to attract female mates. While non-human chorusing appears cooperative to the extent that inter-individual coordination maximizes the intensity and geographic reach of the collective broadcast, these communal displays can arise via competitive mechanisms through which individual males jam rival signals (Greenfield, 1994, 2005). By extension, the selective enhancement of the singer's formant observed in our study suggests that, in human music, an individual beacon motivated by competition can safely nest within a group beacon arising through cooperation.

Apart from enhancing the singer's formant in basses, female presence did not otherwise impact reliably upon performance. Features including balance in terms of overall vocal intensity, global tempo, local tempo fluctuations, and degree of temporal coordination between voices were generally commensurate across performances with and without female audience members. This indicates that individual singers did not attempt to attract attention by obvious means such as producing louder or earlier sounds than their co-performers—unlike the case in non-human chorusing. Clearly, the tradition of excellence and discipline in the St. Thomas Choir and the presence of a conductor (who was blind to the manipulation) precluded variations in rhythmic precision or vocal blend that would compromise ensemble cohesion. Indeed, the fact that we observed any vocal embellishment at all is remarkable given that the music was composed for religious celebration in the Lutheran Protestant tradition, where flamboyance is eschewed (in contrast to opera, gospel, and pop choirs, for example).

The absence of effects for measures of ensemble timing and balance also argues against the possibility that the enhancement of the singer's formant for the second of three performances represents a practice effect. Aspects of performance that are central to the explicit goal of achieving ensemble cohesion, such as timing and balance, are presumably more likely to be susceptible to practice effects than unsanctioned behaviors such as the observed spectral modifications. In any case, practice effects were not expected to occur with the St. Thomas Choir due to their extensive experience with the material performed. Fatigue effects were likewise not expected due to the choir's routine experience singing much longer programs, including masses and oratorios lasting several hours.

As noted earlier, our approach entails a single case study of an elite musical ensemble rather than a fully controlled experiment addressing general music making in a random sample of individuals. Conducting a case study with experts allowed us to provide a hard test of the hypothesis that female presence selectively impacts upon spectral rather than temporal or intensity aspects of male vocal production, and that this effect is specific to males who have undergone voice changes associated with puberty. These goals would not be readily achieved with alternative study designs. Amateur choirs typically comprise sections in which individuals are mixed in terms of age and experience (especially amongst tenors and basses), making these variables difficult to place under experimental control. Forming an *ad hoc* choir for the purpose of the study would likewise be problematic due to the fact that new ensembles usually spend considerable time—several weeks of regular rehearsal—learning to produce a cohesive sound. Moreover, true ensemble excellence is a hallmark of years of working together (Murnighan and Conlon, 1991; Blank and Davidson, 2007). A choir lacking such extensive preparation would most likely produce a high degree of variation in performance timing and balance, which could mask subtle spectral effects associated with the singer's formant.

Of course, investigating a single elite group has the obvious limitation of a small sample size. For this reason, caution should be taken in drawing generalized conclusions, especially since the current study focused on a single, culture-specific musical style. Indeed, one may ask whether effects observed with a highly drilled European boys choir would generalize to everyday forms of group music making in different cultures. While we cannot answer this question based on present findings, work in the field of ethnomusicology suggests that such generalizability is feasible. Group singing is vital to the existence of many indigenous cultures. For example, the male Amazonian Mekranoti Indians sing daily before dawn in a defensive vigil that maintains arousal to guard against attacks from neighboring tribes (Werner, 1984; Huron, 2001) while the nearby Suya people use song as a routine communicative device that complements and overlaps with speech (Seeger, 1987). It is possible that acoustic analyses of these everyday group vocal displays would reveal spectral modulations based on social communicative goals.

Notwithstanding caveats related to the case study approach, our results have several implications for future studies aimed at understanding human non-verbal communication from the perspectives of evolutionary biology, psychology, music cognition, and bioacoustics. With regard to the evolutionary biology, the concept of simultaneous cooperation and competition points to a relationship between human and non-human “chorusing” that goes beyond the fact that both involve rhythmic coordination. Building on this link, the hypothesis that the evolutionary benefit of music lies in balancing sexually motivated behavior and group cohesion advances theory by bringing together opposing accounts of the origins of music, thus highlighting a promising avenue for cross-species comparative research. With regard to psychology, our study highlights music's potential to advance knowledge about the dynamics of non-verbal communication during social interaction. In particular, demonstrating how cooperative and competitive mechanisms operate in parallel—when differing goals are pursued at the level of the group and the individual—showcases music as an ecologically valid domain in which to investigate this social balancing act under controlled conditions (D'Ausilio et al., 2015).

Finally, with regard to music cognition and bioacoustics, the current study is informative about the role of sound quality, in particular the singer's formant, in communicating performers' intentions. In addition to its well-documented function in adding carrying power to the voice in operatic soloists (Sundberg, 1987), our results suggest that the signer's formant may be used to draw the listener's attention to a particular auditory stream in a multipart choral texture. It is important to note that this effect was observed in an elite ensemble under conditions that ostensibly favor the blending of voices. This emphasizes the need for models of musical expression, which to date focus predominantly on variations of timing and intensity (Todd, 1985, 1992; Clarke, 1988; Palmer, 1997; Gabrielsson, 2003; Keller, 2012), to take into account the role of spectral properties in signaling performers' intentions in ensemble music.

In conclusion, the findings of the present study suggest that, analogously to non-human chorusing, human music making may enable the coexistence of cooperative and competitive behavior that potentially mediates social communication simultaneously at the level of the group and the individual. Even during highly formalized types of musical interaction (exemplified here by Western church music), performers are free to introduce unsanctioned behavioral embellishments that allow individuals to enter into covert competition for the attention of potential mates without undermining collaborative goals related to emotional communication and artistic expression. The fact that we observed such subtle modifications in the singing voice—an ancient and universal means of musical expression (Lomax and Berkowitz, 1972; Nettl, 1983; Mithen, 2005; Hagen and Hammerstein, 2009)—raises the possibility that balancing sexual competition with group cohesion in musical contexts is a human capacity with deep biological roots. Accounts of the origins of human music in social cohesion vs. sexual selection can therefore be unified in the hypothesis that the evolutionary benefit of music lies in its ability to serve cooperative and competitive functions simultaneously, with this duality augmenting music's communicative power.

## Ethics statement

This study was carried out in accordance with the recommendations of the ethics committee of the University of Leipzig with written informed consent from all subjects. All subjects gave written informed consent in accordance with the Declaration of Helsinki. The protocol was approved by the ethics committee of the University of Leipzig.

## Author contributions

RK and PK designed the study with input from GN. RK and PK collected and analyzed the data. All authors discussed the results. PK wrote the manuscript with input from RK and GN.

### Conflict of interest statement

The authors declare that the research was conducted in the absence of any commercial or financial relationships that could be construed as a potential conflict of interest.

## Supplementary material

Supplementary material includes audio recordings of the full choir's three performances of the two pieces:

Audio S1 chorale_1_females_absent.mp3

Audio S2 chorale_2_females_present.mp3

Audio S3 chorale_3_females_absent.mp3

Audio S4 fugue_1_females_absent.mp3

Audio S5 fugue_2_females_absent.mp3

Audio S6 fugue_3_females_absent.mp3

Note that the 16 separate channels (one per choir member) are mixed in these files to give an overall impression of the choir's performances, and the files have been compressed for presentation purposes. Uncompressed files are available upon request from PK (p.keller@westernsydney.edu.au).

## Appendix

Note that this questionnaire has been translated into English from the German original used in the study.

**Questionnaire**

**Please enter your number here: ________**

**Age: ______**

**Voice group: ______________________________**

1. How well were you able to concentrate *today* while singing?
2. On who or what do you concentrate generally while singing?(multiple responses permitted)
  o Own voice        o Overall choral sound
  o Neighbors         o Conductor
  o Audience          o Other:_____________________
3. Do you sometimes concentrate on the audience during concerts?
4. Did the audience distract you while singing *today?*
5. What changes did you feel in yourself while the school students were present? *(multiple responses permitted)*
  o I tried to concentrate more on my singing
  o I generally concentrated more than usual
  o I was nervous
  o I tried to attract the students' attention
  o I tried to sing louder
  o I did not experience any changes
  o Other: _____________________
6. In your opinion, which part (block) went best for all of you today?
  o Part 1     o Part 2     o Part 3
